# The Wonderful Drug Clerk

**Published:** 1887-02

**Authors:** 


### The Wonderful Drug Clerk.

The most satirical shot at illegible prescriptions that we have yet seen is the following from the *Medical Age:*

A gentleman received a note from his lawyer which he was unable to decipher. On his way to his office he met a friend, at the door of a drug store. The friend after vainly attempting to read the note suggested that they step inside and hand it to the druggist without comment. The druggist after studying it in silence for a few minutes stepped behind the prescription case, and in a short time returned with a bottle of medicine, duly labeled and bearing directions. When the gentleman saw his lawyer, he was informed that the note was a notice for him to call at his office between three and four the following day. It is a pretty difficult matter to "stick" the regulation druggist.—*Med. Age.*

A VERY interesting communication to the *Medical News* has been made by Dr. F. Peyre Porcher of Charleston, on the influence of the recent earthquake shocks in that city upon the health of the inhabitants. In addition to the natural alarm and fright which were quite universal, some persons were attacked with nausea and vomiting, which recurred or persisted in several cases for days. Two gentlemen on the islands eighty miles from Charleston had their eyes filled with tears not to be repressed, but not caused by alarm, or fears for their personal safety, for the danger there was not imminent. Many persons experienced decidedly electrical disturbances, which were repeated upon the successive recurrence of the shocks. These were generally tingling, pricking sensations, like 'needles and pins,' affecting the lower extremities. One gentleman was completely relieved of his rheumatism; another, who for months was nervous, depressed, and entirely unable to attend to business, regained his former activity and energy. Several cases of mental disturbance, owing to anxiety and prolonged loss of rest, some of them persistent, occurred among Dr. Porcher's patients.

Dr. Giles de la Tourette finds that the average step of men is twenty-five inches; for women, twenty inches. The step with the right foot is somewhat longer than that with the left. The feet are separated laterally in walking about four and one-half inches in men, and five in women.

The Young-Helmholtz theory of color-sensation has recently been put to the test of direct experimental proof by Herr Frithiof Holmgren (*Verhandlung der physiolog. gesellschaft zu Berlin*, 1886, No. 18). As is well known, the theory is that the retina contains three sets of nerve-elements, each set capable of responding to the stimulus of a single color alone; and that the three colors which correspond to three sets of nerve-elements are green, red, and violet. These are the primary colors, and our sensation of all others is due to the simultaneous excitation of nerve-elements of different sets. Now, it is possible to produce a point of light so minute that its image on the retina shall have no greater dimensions than those of a single nerve-element or cone. If such a point of light in any color of the spectrum be examined in such a way that its image falls in turn upon different parts of the retina, it will, if the Young-Helmholtz theory be true, be seen only as red, green, or violet. If one of these primary colors be chosen for examination, it will appear in its own shade or not at all; but, if any other shade is employed, it will be resolved into its primary elements, and seem red, green, or violet, according to its composition and the particular cone on which it falls. The results of Holmgren's investigation were in entire accordance with the theory; red, green, and violet (indigo-violet) were unchanged; yellow appeared red, green, or colorless, in no part of the field destinctly yellow; blue was resolved similarly into green and violet. Further experiments, with a view to determining how many cones must receive simultaneous stimulus to produce the sensation of a particular color, show that yellow is seen as red or green even when the retinal image is considerably smaller than the section of a cone; while, to be seen as yellow, the image must be large enough to cover two or three cones.

The fallacy contained in the common saying that numbers cannot lie, is well shown in the recent discussion of the statistics of insanity by Dr. D. Hack Tuke. The statistics may be all right, but they must be taken in a certain way to warrant definite conclusions. From the facts that more cases of insanity are now treated, that we have more asylums, and that our age is called a neurotic one, the mournful conclusion is drawn that a greater proportion of civilized humanity is succumbing to the stringent requirements of modern life, and losing its mental equilibrium. Dr. Tuke shows, that, by such statistics, the insane of the past thirty years or so, whose lives our improved methods of treatment have succeeded in prolonging, are pushed upon our shoulders. The real test of the prevalence of insanity is the proportion of first attacks occuring within certain periods. On this basis, Dr. Tuke shows that since 1878 (the earliest date from which adequate statistics exist) there is no increase in *occurring* insanity in Great Britain. On the whole, there is a slight tendency to decrease; and this, too, though cases are now more apt than ever to be brought to notice. Of course, this should not lessen our vigilance in the matter, nor remove our attention from that large class on the borderland of insanity which is not recorded, and from which any sudden crisis chooses its victims.

The physiology of digestion has been so thoroughly investigated of late years, that it would seem that there could be very little opportunity for difference of opinion on most of its leading principles, and yet we find that authorities are on some points very much at variance. We are told that nothing can be more prejudicial than the habit of chewing gum, supposed to be so common among school-children. The salivary glands are unnaturally excited, and pour forth so much salva in the act, that when food is masticated they are not able to respond as fully as is necessary for the proper insalivation of the food. We are also informed that food should not be eaten just before retiring; that thoroughly refreshing sleep requires perfect repose of all the organs; and that, if we go to sleep with a more or less full stomach, sleep will be disturbed and unsatisfactory. The authorities of Amherst college evidently do not agree with these views. In the instructions which they give to their students to guide them in their gymnastic exercises, after specifying the kind and amount of physical exercise, they recommend sleeping for half an hour after dinner and supper if possible, and, if sleepless at night from brain-work, eat a few graham crackers before retiring, to draw the excess of blood from the brain to the stomach. In reference to the practice of chewing gum, this statement is made; chewing gum daily before eating and between meals increases the flow of saliva, and so aids the digestion of fat-making foods. It also indirectly stimulates the secretion of the digestive juices of the stomach. We have no means of knowing, but we presume that professor Hitchcock of Amherst, who is himself a physician, is largly responsible for this advice, and have no doubt that he has given it after mature consideration. We fully agree with what is said in the instructions about the usefulness of food in cases of sleeplessness, and believe that many a person has been kept awake at night from a mistaken idea of the necessity of abstemiousness before retiring. This, of course, does not mean that late suppers are under all circumstances to be recommended; but a few graham crackers can never do harm, and will often do good. In regard to the chewing-gum, we do not feel so sure. Besides being a practice which is from an aesthetic point of view not to be encouraged, it is very doubtful whether, under the most favorable circumstances, it is really a benefit to digestion; and, until there is some guaranty as to the composition of what is called chewing-gum, we should hesitate before recommending it in such unqualified terms.—*Science.*

